# Decreased postural control in adult survivors of childhood cancer treated with chemotherapy

**DOI:** 10.1038/srep36784

**Published:** 2016-11-10

**Authors:** Einar-Jón Einarsson, Mitesh Patel, Hannes Petersen, Thomas Wiebe, Per-Anders Fransson, Måns Magnusson, Christian Moëll

**Affiliations:** 1 Department of Clinical Sciences, Lund University, Lund, Sweden; 2 Faculty of Medicine, University of Iceland, Reykjavik, Iceland; 3 Division of Brain Sciences, Imperial College London, London, United Kingdom; 4 Department of Otorhinolaryngology, Landspitali University Hospital, Reykjavik, Iceland; 5 Department of Paediatrics, Skåne University Hospital, Lund, Sweden; 6 Department of Otorhinolaryngology, Skåne University Hospital, Lund, Sweden

## Abstract

The objective of cancer treatment is to secure survival. However, as chemotherapeutic agents can affect the central and peripheral nervous systems, patients must undergo a process of central compensation. We explored the effectiveness of this compensation process by measuring postural behaviour in adult survivors of childhood cancer treated with chemotherapy (CTS). We recruited sixteen adults treated with chemotherapy in childhood for malignant solid (non-CNS) tumours and 25 healthy age-matched controls. Subjects performed posturography with eyes open and closed during quiet and perturbed standing. Repeated balance perturbations through calf vibrations were used to study postural adaptation. Subjects were stratified into two groups (treatment before or from 12 years of age) to determine age at treatment effects. Both quiet (p = 0.040) and perturbed standing (p ≤ 0.009) were significantly poorer in CTS compared to controls, particularly with eyes open and among those treated younger. Moreover, CTS had reduced levels of adaptation compared to controls, both with eyes closed and open. Hence, adults treated with chemotherapy for childhood cancer may suffer late effects of poorer postural control manifested as reduced contribution of vision and as reduced adaptation skills. These findings advocate development of chemotherapeutic agents that cause fewer long-term side effects when used for treating children.

The maintenance of postural control in humans requires continuous integration of sensory inputs from visual, vestibular and somatosensory receptors (proprioceptors and mechano-receptors) by the Central Nervous System (CNS) to assess position and motion of the body^1^. The fundamental mechanisms behind postural control (e.g. sensorimotor development and functional ability) are formed and modified between childhood and adolescence through physical activity^2^. The developmental process of postural control involves several phases such as building repertoires of different postural strategies and learning how to apply these strategies to different environments through exploration^3^,^4^. Another process is learning to anticipate events and selecting the most appropriate strategy^5^. Children with conditions that affect these processes may never master complex motor repertoires.

The treatment of childhood cancer often requires a concoction of different chemotherapeutic agents of varying quantities. However, chemotherapeutic agents are destructive in nature and could lead to poor postural control and sensorimotor development, either through direct action (i.e. by impairing sensory or motor functions) or indirectly (i.e. side-effects such as fatigue or nausea which may render the child bedridden for extended periods of time). For example, motor problems including postural instability have been found in children 2–7 years after completion of chemotherapy where vincristine has been part of the treatment^6^,^7^,^8^. Vincristine can cause decreased peripheral muscle strength^6^, disrupt control of ankle dorsiflexion^9^,^10^ and cause polyneuropathy which may lead to lower leg sensory loss^11^. Reports suggest that the late effects from vincristine are influenced by the child’s age at treatment, with early infants most heavily affected^12^. Moreover, vincristine and methotrexate can cause neurotoxicity^13^,^14^ but these effects may first appear several years after treatment^15^. Another chemotherapeutic agent known to have detrimental effects on children is cisplatin, which is both neurotoxic^16^ and ototoxic and may damage the vestibular and auditory systems^17^,^18^,^19^. Subsequently, ototoxicity can potentially result in dizziness and disequilibrium^20^.

Significant work has been carried out to study the long-term effects of chemotherapy in children^15^,^21^ but no previous studies have considered the effects on adaptive postural control which is fundamental to meet the pressures of physical environments. As chemotherapeutic agents administered in childhood could damage the sensory and motor systems, an improper development of these systems in survivors of childhood cancer may lead to long-term postural instability. Such deficits are more likely to be revealed when stability is challenged^22^. Vibration of skeletal muscles or tendons is a common method used to controllably perturb balance and assess an individual’s ability to maintain a stable stance^23^. When repeated, the brain generates sensorimotor predictions about the likely outcome of the event and accordingly adapts our motor plans^24^. This is an error based motor learning process that quickly allows modification of motor strategies to maintain postural control^4^. Sensorimotor adaptation is an essential feature of postural control since it decreases fall risk^25^ - normal individuals should quickly be able to learn the characteristics of a balance challenge and set their balance system to minimise these effects^4^.

The aim of this study was to investigate postural control and adaptation in adult survivors of childhood cancer treated with chemotherapy. To study the long-term effects of chemotherapy on postural control, the subjects were assessed on average 15 years after end of treatment. The subjects recruited had been treated for malignant solid tumour types commonly found in children and had been treated with common types of chemotherapy medication. Strict selection criteria for the malignancy were used to avoid confounding peripheral or central nervous system involvement. Another aim was to determine if the age at the time of chemotherapy treatment influences postural stability. This investigation is part of a set of studies in which adult populations, treated for cancer with chemotherapy at young age, have been screened for late subjective and neurological symptoms^18^,^19^,^26^.

## Results

### Quiet stance stability

There was no significant difference between CTS and healthy controls for quiet stance postural stability (ANOVA, Table 1). Vision significantly increased stability in both subject groups, i.e., reduced used total (p < 0.001), low frequency (p = 0.034) and high frequency energy (p < 0.001). There was no evidence of interaction between having chemotherapy and vision.

**Figure 1:**
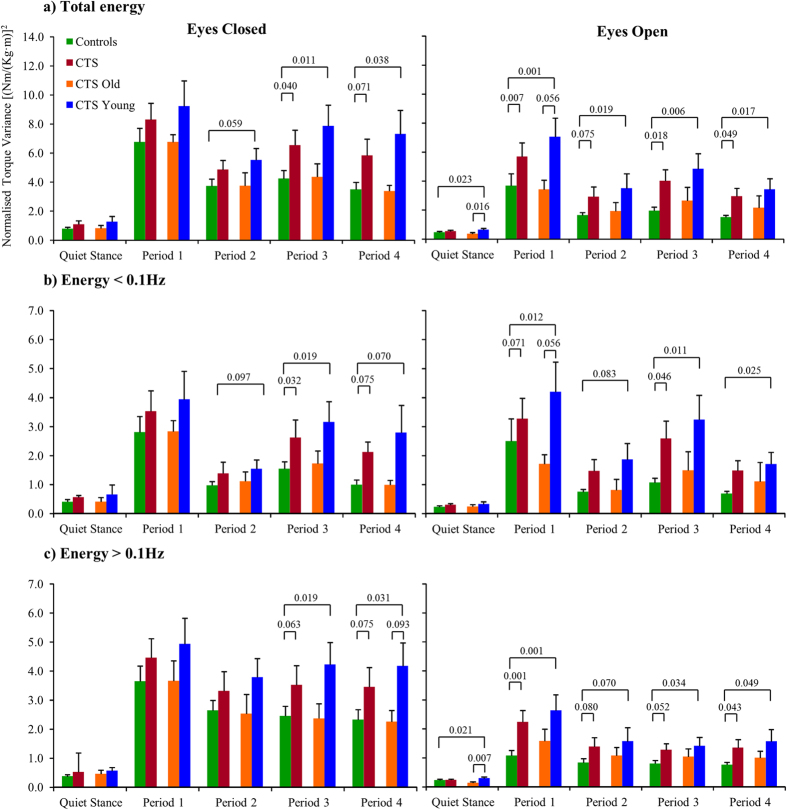
Total, low frequency and high frequency spectral energy used towards the supporting surface during the 5 sequential time periods during posturography. The figure present normalised; (**a**) total, (**b**) low frequency and (**c**) high frequency torque variance values, representing kinds of spectral energy, used during posturography while standing with Eyes Closed and Eyes Open (mean and SEM). Values and statistical findings to the level of trends (p < 0.1) are presented for healthy controls (n = 25), for all chemotherapy treated subjects CTS (n = 16), and for the subgroups CTS treated with chemotherapy below 12 years of age CTS_Young (n = 10) and CTS treated from 12 years of age and older CTS_Old (n = 6).

Sub-group analyses showed that CTS_Young had poorer stability compared to controls and CTS_Old. CTS_Young used more total energy in quiet stance than controls (p = 0.040). Moreover, CTS_Young used more high frequency energy than CTS_Old (p = 0.049). Post-hoc analysis confirmed that CTS_Young had used more total and high frequency energy with eyes open compared to controls (p ≤ 0.023) and CTS_Old (p ≤ 0.016) (Fig. 1).

### Perturbed stance stability

During balance perturbations, CTS had significantly poorer stability compared to controls as reflected by higher use of total (p = 0.012), low frequency (p = 0.024) and high frequency energy (p = 0.019) (ANOVA, Table 2). In the ANOVA, vision significantly increased stability in CTS and controls i.e., used total (p < 0.001), low (p = 0.049) and high frequency energy (p < 0.001) were lower with eyes open compared to closed. CTS and controls showed significant adaptation (ANOVA p < 0.001 for all frequency spectra) i.e., a reduction of used total, low frequency and high frequency energy to address repeated perturbations across Periods (i.e., Periods 1–4). In the investigation of interactions between main factors, CTS used more high frequency energy over time (i.e., in Periods 3 and 4) than controls (p = 0.017) and especially with eyes closed (p = 0.022), see also Fig. 1.

When studying age at treatment effect, CTS_Young had poorer stability compared to controls in all spectral categories (p ≤ 0.009). Main factor interactions revealed that energy used over time increased during posturography in CTS_Young compared to controls (p = 0.042), that the pattern of energy used over time were systematically different between eyes closed and open (p = 0.016) and that the CTS_Young used more energy over time compared to controls with eyes closed (p = 0.008). When comparing the CTS_Old and controls the stability was similar. However, when comparing CTS_Young with CTS_Old, CTS_Young used significantly more high frequency energy especially in the last periods of tests with eyes closed (p = 0.045).

Post-hoc analysis confirmed poorer stability during balance perturbations in CTS compared to controls for Period 3 in total and low frequency energy with eyes closed (p ≤ 0.040); and with eyes open during Period 1 in total and high frequency energy (p ≤ 0.007), Period 3 in total and low frequency energy (p ≤ 0.046) and Period 4 in total and high frequency energy (p ≤ 0.049) (Fig. 1).

When studying age at treatment effect, CTS_Young had poorer stability with eyes closed than controls and the differences were accentuated over time especially as increased total and high frequency energy used during vibration Periods 3 and 4 (p ≤ 0.038). However, CTS_Young also had poorer stability with eyes open compared to controls for total energy in Periods 1-4 (p ≤ 0.019) and in low and high frequency energy during Periods 1, 3 and 4 (p ≤ 0.049). In post-hoc tests, there was no significant difference between CTS_Old and controls.

### Adaptation capacity

When investigating the ability to adapt to the repeated balance perturbations between Period 1 and Period 4 (i.e., the maximum level of learning), controls showed significant adaptation in all frequency spectra with eyes closed (p < 0.001) and eyes open (p ≤ 0.002) (Table 3). CTS also showed significant adaptation in all frequency spectra with eyes open (p ≤ 0.005) but only in the total and low frequency spectra with eyes closed (p ≤ 0.006), not in the high frequency spectra. The level of adaptation was poorer in CTS compared to controls.

A further analysis of the effect of age at treatment revealed that CTS_Young had no significant adaptation to balance perturbations with eyes closed and a significant adaptation limited to total and low frequency spectra with eyes open (p ≤ 0.004). CTS_Old had a significant adaptation limited to total and low frequencies with eyes closed (p ≤ 0.031), and only in the high frequency spectra with eyes open (p = 0.031).

## Discussion

The primary objective with cancer treatment is to secure the survival of the patient. This primary objective has increasingly been achieved in the last few decades by the introduction of new, more effective treatments, which have substantially improved survival rates especially when treating children and adolescents. However, this success has highlighted the importance of monitoring long-term effects from the cancer and from the treatments used in order to improve the overall quality of life in cancer survivors, particularly for neurodevelopment^21^. Considering the long time span during which side-effects from chemotherapy may develop, a specific objective of this study was to investigate for impairments in postural control and sensorimotor adaptation in adult survivors of childhood cancer. For example, neuropathy, a known neurotoxic side effect from vincristine and methotrexate^13^,^14^, may appear first several years after the end of treatment^15^. Moreover, ototoxic drugs induce a gradual loss of sensory cells and primary vestibular neurons^17^,^20^, which may develop into a significant functional lesion many years after the end of treatment. This study revealed that adults treated with chemotherapy in childhood have poorer postural control compared to controls, on average 16 years after the end of treatment, which is consistent with other reports^6^,^27^. Moreover, the stability issues were manifested as similarly increased use of energy in all spectral categories, i.e., both as more smooth corrective changes of posture (i.e. <0.1 Hz) and more fast reflexive corrective movements to maintain balance (i.e. >0.1 Hz), suggesting that the deficits in CTS affects several sensorimotor systems^28^,^29^,^30^,^31^. Another new finding in this study is that subjects treated at younger age are more likely to experience poor postural control as an adult.

Importantly, this study suggests that the developmental state of being a child or adolescent at the time of treatment might be a critical factor influencing the severity of any related impairment into adulthood. The postural control deficits were found to be significantly more severe among those treated at younger age, affecting both the ability to utilise vision and adaptation to enhance postural control during balance perturbations. It is possible that the immaturity of the peripheral and central nervous systems in children made these systems more susceptible to physical and functional changes and irreversible damage from chemotherapeutic agents^20^. Another possibility is that if severe peripheral or central impairments are received before the developmental stages of sensory and motor systems are complete, these systems may never fully recover through central compensation. A limitation of this study is that tests were not carried out repeatedly from the end of treatment to map systematic changes in performance over time. However, our findings are a strong indication that longitudinal assessments should be performed regularly on subjects treated in childhood with chemotherapy for at least 20 years after the end of treatments.

The visual system usually plays an important role in the maintenance of postural stability, especially when information from other sensory receptors is unreliable. The present study showed that during perturbations, visual information (eyes open) improved stability in CTS compared with absent visual information (eyes closed). However, the level of improvement of stability when utilising vision was lower compared to controls. Interestingly, problems associated with the visual system have been found in chemotherapy treated subjects in previous psychophysical and questionnaire investigations^26^, where difficulties with visual tasks were identified by CTS themselves^26^. Thus, the similarities in objective and subjective measurements are compelling regarding this matter. Experimental alterations in visual performance (i.e. clarity and acuity) are known to decrease postural stability in eyes open tests^32^,^33^, and a number of vestibular disorders can impair oculomotor control, causing oscillopsia^34^. Hence, reduced or altered vestibular and/or visual performance could partly account for the poorer stability in CTS. It is possible that the CNS is either not able to fully utilise the visual information properly to enhance postural control or that the spatial and/or temporal information provided by the visual system is less reliable in CTS.

Subjects treated as children with chemotherapy had impaired sensorimotor adaptation of postural control evidenced by poor motor learning to repeated perturbations, both when standing with eyes closed and open. Specifically, for CTS_Young, adaptation without vision proved challenging. The explicitly poorer than normal performance during the last two 50-second periods of the posturography tests suggest that a contributing factor might be that repeated perturbations for over 2 minutes became increasingly physically and/or mentally fatiguing. Moreover, using positron emission tomography, Silverman *et al*.^35^ found alterations in resting metabolism and cerebral blood flow in the basal ganglia, inferior frontal gyrus and cerebellum during memory activation in women who had received chemotherapy 5–10 years earlier. These CNS areas are all associated with sensorimotor adaptation. In addition, several studies report hippocampal atrophy as a post-effect from chemotherapy^36^,^37^,^38^. Decreased adaptive capacity might also be caused by decreased peripheral sensory information, which may hinder the CNS from getting the information needed to allow an effective adaptive process^39^. Known side-effects from chemotherapy agents include polyneuropathy, which may cause decreased somatosensory sensation, and ototoxity which may produce vestibular lesions^11^,^16^,^17^. Thus, if chemotherapy results in impairment of adaptation, it may limit the efficacy of rehabilitation and training programs^40^. In a subgroup of our material, signs of ototoxity were explored by comparing hearing levels before chemotherapy and at the time of posturography, on average about 16 years later. These assessments revealed substantial hearing losses in 4 out of 10 investigated subjects. However, when statistically evaluated, no finding or trend suggested more impaired postural control among those who had suffered hearing loss.

Possible causes for long-term impairments after childhood cancer may not only include the chemotherapy treatment received but may also include lesions produced by the cancer itself or be a consequence of the intervention treatment (surgical removal and radiation). Strict criteria for the malignancy were therefore applied in this study to avoid confounding peripheral or central nervous system impairment from the cancer itself, radiation or surgery treatments. Another consideration is that the cancer and treatments might make the child fatigued or nauseated, which may render the child bedridden and inactive for extended periods of time during a part of their life where many of the physical and sensory systems are still in a process of development. This may postpone the natural development of motor skills^2^.

Nevertheless, due to severity and range of side-effects experienced many years after chemotherapy treatment and the growing awareness of relationships between chemotherapeutic agents and a multitude of long-term adverse effects^16^, the most likely cause for long-term impairments after childhood cancer is the chemotherapy treatment received. Chemotherapy is generally not very specific, and it puts normal tissues and organs at risk. Although the brain is given some protection from systemic treatments by the blood-brain barrier, it is increasingly recognized that many chemotherapeutic agents affect central brain function through direct and/or indirect mechanisms^41^. Moreover, the long-term consequences of most chemotherapeutic agents on the CNS have rarely been considered^42^. However, Dietrich and colleagues have recently showed that clinically relevant concentrations of 1,3-bis(2-chlorthyl)-1-nitrosoura (BCNU) and cisplatin, are even more toxic to CNS progenitor cells and oligodendrocytes than they are to cancer cell lines^43^. Furthermore, therapeutic levels of 5-fluorouracil (5-FU) is associated with progressive delayed damage to myelin^44^.

The objective of this study was not to determine the long-term effect of specific chemotherapy drugs but to investigate if subjects in general with this medical history suffer from postural instability and reduced adaptation even 15 years after treatment. However, this study illustrates the complexity of determine the effects of individual chemotherapy agents. As shown in Table 4, the treatment of childhood cancer often requires a concoction of different chemotherapeutic agents applied in varying quantities and over different durations. Hence, large-scale, long-term studies are likely necessary to identify drug-associated side-effects, e.g., whether certain drugs are more toxic than others and under what circumstances impairments from chemotherapy treatment in children occur. This information would though be markedly useful in order to detect, prevent and treat the impairments from chemotherapy treatment.

Previous reports have largely implicated vincristine as the causative agent for impaired postural control reported in chemotherapy treated subjects several years after the end of treatment^6^,^11^,^45^, though the effect of other drugs such as methotrexate^13^,^14^ and cisplatin^17^ should not be ruled out. Furthermore, a strict causal relationship between cumulative vincristine dose and motor performance has not yet been proved, although low doses of vincristine have been found to decrease motor control^6^. Moreover, many chemotherapeutic agents including vincristine, cisplatin and carboplatin affect the eye periphery and subsequent sensory information, which could result in grey-matter atrophy in the occipital lobe^16^. However, it is particularly interesting that vincristine induces neuropathy to cranial nerves, especially of the ocular motor nerves^46^,^47^. Any structural brain changes in children could interfere with normal development resulting in abnormal function of the affected area^48^.

It is also important to consider to what extent chemotherapy treated subjects suffer from various subjective and neurological symptoms causing reduced quality of life and where some symptoms may be associated with impoverished postural control. We have previously investigated oculomotor and neurological status, and subjective symptoms in a somewhat larger study population in which the subjects participating in this study also took part^26^. We noted a similar trend as in this study that subjects treated before 12 years of age experienced greater impairments from the chemotherapy treatments received. The study also found among the test subjects marked oculomotor control deficits and the subjects reported in a questionnaire frequent issues with visual disturbances, dizziness, light-headedness and perceptual unsteadiness. Taken together, the current findings and our previous findings suggest that several basic neurological functions and everyday actions are often impaired in adults treated with chemotherapy, specifically in adults treated with chemotherapy before 12 years of age.

Due to strict inclusion criteria subgroup numbers are small, which might affect the reliability of the subgroup statistical analyses. Moreover, there were clear gender, height and weight differences between subgroups CTS_Young and CTS_Old. However, the lower weight and height found for CTS_Young are usually associated with better postural control, thus, the age at treatment related findings are not related to differences in anthropometrics. Furthermore, the different ability to use vision to enhance stability is difficult to explain by anthropometrical differences between CTS_Young and CTS_Old.

Because of the strict inclusion criteria applied, the subject population investigated were stringently defined in terms of cancer type category (i.e., malignant solid tumours). Moreover, the inclusion criteria were carefully selected to ensure that the chemotherapy treatment received would be the only likely source for any late effects observed. However, the strict criteria applied made us in the end include only 16 subjects out of approximately 750 patients in the total cohort investigated. Hence, since this study has validated the concept and determined a marked practical clinical relevance in a very strictly controlled material, a natural development of the research would be to perform prospective investigations carried out on larger and more homogenous populations, e.g., where all subjects had suffered from the same type of cancer (e.g., leukaemia in childhood) and where the treatment procedures were more standardised. Moreover, future large cohort studies may also approach the complex but vital issues of childhood cancer treatments such as determining potential influences of cancer types, cancer locations, chemotherapy agents received, medication administration procedures and the sizes of accumulated medication dosages.

To conclude, neurotoxic signs and symptoms tend to decrease over time as a result of central adaptation^6^,^49^. However, we found poor postural control and adaptation on average 16 years after the end of treatment in CTS subjects, suggesting poor ability in this patient population to recover from chemotherapy treatment side effects. These findings emphasize the necessity to follow up, also long-term into adulthood, cancer patients treated in childhood with chemotherapy and to introduce early in the cancer treatment recovery programs also balance rehabilitation regimes. Moreover, the findings advocate development of chemotherapy agents that cause fewer and less harmful long-term side effects when used for treating children.

## Methods

Experiments were performed in accordance with the Helsinki declaration and approved by the Scientific Ethical Committee at Lund University, Sweden (number LU964-03) and by the Data Protection Authority (number LU-P6103), Sweden. A non *obstat* statement was obtained from the Scientific Ethical Committee, that no additional ethical approval was required to perform the investigations as part of clinical follow-up of the patient population. All participants or their guardians, provided written informed consent before the testing commenced.

### Subjects

Forty-one subjects, 16 chemotherapy treated subjects (CTS) and 25 healthy controls participated in the study. The CTS were recruited from all adults who survived childhood cancer in the county of Skåne, Sweden, between 1980 and 2000. Sixteen of approximately 750 adult survivors from the period, fulfilled the strict inclusion criteria: cancer diagnosed before the age of 18; treated for a malignant solid tumour not affecting CNS with chemotherapy agents and the treatment completed >5 years before this study. Exclusion criteria from the study were if the subjects had received cranial radiotherapy or surgery which might have affected the CNS or had surgery affecting the legs or pelvis. Subjects who fulfilled all criteria were contacted by a clinical administrator and offered to participate in the study, which they all did. The investigations were performed without any selection bias.

The 16 CTS (7 females; mean age 26.1 years (SD 6.9); mean height 1.73 meters (SD 0.10); mean mass 71.2 kilograms (SD 18.6)) were on average 10.1 years (SD 5.7) old when submitted to chemotherapy treatment and the subjects had on average completed the chemotherapy treatment 16.1 years (SD 5.8) before participating in this study. Additional treatment details are presented in Table 4. Ten of the sixteen subjects in this study underwent clinical hearing assessment before receiving chemotherapy, and thus, the changes in hearing level could be determined in these subjects at the time of posturography testing, see Table 4. When rating the hearing loss with Brock’s hearing loss grades, four of ten subjects had suffered a substantial hearing impairment during and after the chemotherapy treatment whereas the other six subjects had preserved normal hearing.

To determine whether the developmental state at treatment influenced postural control and sensorimotor adaptation, the CTS group was also divided into two age subgroups. One subgroup (CTS_Young), included all CTS that received treatment before 12 years of age (n = 10; 6 females; mean age 24.2 years (SD 7.4); mean height 1.70 meters (SD 0.09); mean mass 67.3 kilograms (SD 22.3)) with mean age at treatment of 6.5 years (SD 4.0). The other subgroup (CTS_Old), included all CTS that received treatment from 12 years of age and older (n = 6 subjects; 1 female; mean age 29.3 years (SD 5.0); mean height 1.80 meters (SD 0.12); mean mass 77.8 kilograms (SD 11.2)) with mean age at treatment of 16.0 years (SD 1.0). Statistical analyses showed no difference between subgroups for quantity and type of chemotherapy drugs received.

The age and gender-matched control group consisted of 25 healthy participants (13 women, mean age 25.1 years (SD 4.6); mean height 1.75 meters (SD 0.09); mean mass 68.8 kilograms (SD 13.3)). All subjects included in the study, both controls and CTS, had normal or corrected to normal visual acuity using glasses or contact lenses.

### Posturography assessment

A custom built force platform recorded torques and sheer forces with six degrees of freedom using force transducers with an accuracy of 0.5 N. A customised computer program controlled the vibratory stimulation and sampled the force platform data at 50 Hz. The vibrators had vibration amplitude of 1.0 mm and frequency of 85 Hz, were 6 cm long and 1 cm in diameter and strapped over the calf muscles.

**Figure 2:**
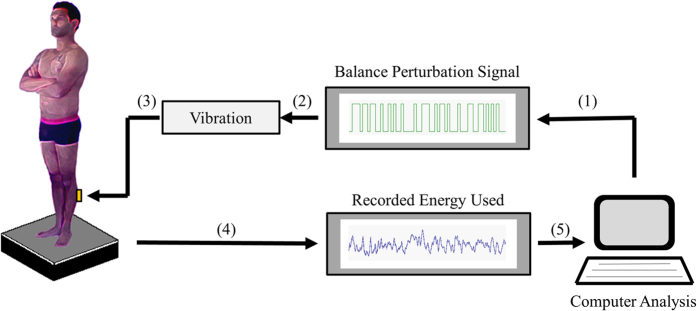
Schematic illustration of the posturography setup. The posturography software produces (1) a pseudorandom binary sequence (PRBS), which is (2) converted to vibration pulses. Vibration pulses applied towards (3) the calf muscles cause balance perturbations. The energy used (4) towards the support surface, reflecting the effort to maintain stability, is recorded by a force platform and quantified by the posturography software (5).

Each participant stood barefoot on the force platform in an upright but relaxed posture with arms folded across the chest, heels 3 cm apart and feet positioned at an angle of 30° along guidelines on the platform, see Fig. 2. Participants were instructed to focus on a target 1.5 m in front of them at eye level or keep their eyes closed depending on the test condition. The participants listened to music through headphones to reduce possible movement references from external noise sources and to avoid extraneous sound distractions. To ensure no prediction of the balance perturbation, all participants were naive to the stimulus and were not informed about the effect calf vibration would have on their balance.

Two tests were performed in a randomised order, using a Latin Square design, by all subjects:

(1) Vibration of the calf muscles with eyes closed (EC) and

(2) Vibration of the calf muscles with eyes open (EO).

Before vibration commenced, a 30 second control period of quiet stance was recorded. The vibratory stimulations were applied according to a pseudorandom binary sequence (PRBS) schedule^22^ during a period of 200 seconds making each trial 230 seconds long. The PRBS schedule defined the periodicity of stimulation pulses, where each pulse and each interval between pulses had random time duration from 0.8 seconds up to 6.4 seconds, which yielded an FFT-validated effective bandwidth of the test stimulus in the region of 0.1–2.5 Hz. The PRBS sequence was selected because this randomised stimulation sequence is difficult to predict and therefore lessens the likelihood of pre-emptive responses. The same PRBS schedule was used across all subjects and for all tests. A five minute rest period was given to the subjects between tests.

### Analysis

Since calf vibration induces body movement mainly in the anteroposterior direction^24^, only the responses in the anteroposterior direction are considered here. Postural stability was measured as the variance of anteroposterior torque values. The torque produced at various sites of the body is the effective means used for controlling the orientation and stability of a standing organism^50^. Also, torque variance values correspond directly to the energy used towards the support surface to maintain stability^51^, which in turn corresponds to the efficiency of standing^50^. For a detailed explanation on torque and its relationship to standing postural control, see Johansson *et al*.^51^.

The force platform recordings were spectrally divided into total torque variance (total energy), torque below 0.1 Hz (<0.1 Hz; low frequency energy); and torque above 0.1 Hz (>0.1 Hz; high frequency energy) using a fifth-order digital Finite duration Impulse Response (FIR) filter, with filter components selected to avoid aliasing. These separations were used to distinguish between smooth corrective changes of posture (i.e. <0.1 Hz) and fast corrective movements to maintain balance (i.e. >0.1 Hz)^28^. Clinically, increased fast corrective movements (>0.1 Hz) are commonly associated with decreased visual information^28^ and by factors influencing motor control such as being over-weight or fatigued^29^, whereas the low frequency movements (<0.1 Hz) are commonly increased by poor surface conditions like standing on foam^30^,^31^. Torque variance values were normalised to account for anthropometric differences between the subjects, using the subject’s squared height and squared weight, as height and weight are key factors influencing the body sway recorded by a force platform^4^,^51^. The squared nature of the variance algorithm made it necessary to use normalisation with squared parameters to achieve unit agreement.

Mean values for all parameters were obtained for five periods for each trial condition: the quiet stance period (0–30 s), and from four 50 s periods (Period 1: 30–80 s; Period 2: 80–130 s; Period 3: 130–180 s; Period 4: 180–230 s) during the vibration. Each 50 s period contains a similar amount of long and short vibration pulses validated by Fast-Fourier Transform (FFT)-analysis of spectral contents in the stimulation. Hence, the selected periods and perturbation sequence allowed analysis of whether the stability changed over time and possibly caused an adaptation to the unpredictable balance perturbations.

### Statistical analysis

The torque variance values during quiet (unperturbed) stance and during balance perturbations were analysed using repeated measures GLM ANOVA on log-transformed values. The main factors and factor interactions analysed were: ‘Chemotherapy’ (CTS (n = 16), CTS_Young (n = 10), CTS_Old (n = 6) vs. Controls (n = 25); d.f. 1); ‘Vision’ (eyes closed vs. eyes open; d.f. 1), and when applicable ‘Period’ (vibration periods 1, 2, 3, 4; d.f. 3).

The Mann-Whitney U (Exact sig. 2-tailed) test was used for between-groups post hoc comparisons. The Wilcoxon matched-pairs signed-rank test (Exact sig. 2-tailed) was used for within-subjects post hoc comparisons, i.e., analysing the adaptive changes over time between Period 1 and Period 4^4^,^24^.

In all analyses, p-values < 0.05 were considered statistically significant, whereas trends were defined as (p < 0.1). Non-parametric statistical tests were used in all statistical evaluations since the Shapiro-Wilk test revealed that some of the obtained data sets were not normally distributed.

## Additional Information

**How to cite this article**: Einarsson, E.-J. *et al*. Decreased postural control in adult survivors of childhood cancer treated with chemotherapy. *Sci. Rep*. **6**, 36784; doi: 10.1038/srep36784 (2016).

**Publisher’s note**: Springer Nature remains neutral with regard to jurisdictional claims in published maps and institutional affiliations.

## Figures and Tables

**Table 1:** Effects of chemotherapy and vision on the stability during quiet stance.

Quiet stance stability	Chemotherapy*	Vision	Chemotherapy x Vision
CTS vs healthy controls			
Total	0.128 [2.4]	**<0.001 [17.6]**	0.832 [0.0]
<0.1 Hz	0.243 [1.4]	**0.034 [4.8]**	0.712 [0.1]
>0.1 Hz	0.278 [1.2]	**<0.001 [30.8]**	0.396 [0.7]
CTS_Young vs healthy controls			
Total	**0.040 [4.6]**	**0.003 [10.2]**	0.571 [0.3]
<0.1 Hz	0.139 [2.3]	0.113 [2.6]	0.519 [0.4]
>0.1 Hz	0.063 [3.7]	**<0.001 [18.4]**	0.837 [0.0]
CTS_Old vs healthy controls			
Total	0.965 [0.0]	**0.002 [11.1]**	0.716 [0.1]
<0.1 Hz	0.886 [0.0]	0.073 [3.5]	0.879 [0.0]
>0.1 Hz	0.571 [0.3]	**<0.001 [27.8]**	0.067 [3.6]
CTS_Young vs CTS_Old			
Total	0.127 [2.6]	**0.012 [8.3]**	0.426 [0.7]
<0.1 Hz	0.385 [0.8]	0.227 [1.6]	0.563 [0.4]
>0.1 Hz	**0.049 [4.6]**	**0.001 [15.6]**	0.135 [2.5]

Repeated measures GLM ANOVA analysis of how the quiet stance stability were affected by main factors “Chemotherapy” and “Vision” alone and by the main factor interaction denoted as “Chemotherapy x Vision”. The notation “<0.001” means that the p-value is smaller than 0.001. F-values are presented in the squared parenthesis.

^*^In the CTS_Young vs CTS_Old GLM Anova evaluation the main factor “chemotherapy” represents the effect of receiving chemotherapy below 12 years of age (CTS_Young) vs from 12 years of age and older (CTS_Old).

**Table 2:** Effects of chemotherapy, vision and vibration period on the stability during balance perturbations.

Perturbation stability	Chemo	Vision	Period	Chemo x Vision	Chemo x Period	Vision x Period	Chemo x Vision x Period
CTS vs healthy controls							
Total	**0.012 [7.0]**	**<0.001 [66.2]**	**<0.001 [125.9]**	0.652 [0.2]	0.095 [2.9]	0.251 [1.4]	0.452 [0.6]
<0.1 Hz	**0.024 [5.5]**	**0.049 [4.1]**	**<0.001 [113.6]**	0.865 [0.0]	0.112 [2.6]	0.334 [1.0]	0.570 [0.3]
>0.1 Hz	**0.019 [6.0]**	**<0.001 [129.3]**	**<0.001 [55.6]**	0.421 [0.7]	**0.017 [6.3]**	0.076 [3.3]	**0.022 [5.7]**
CTS_Young vs healthy controls							
Total	**0.003 [9.9]**	**<0.001 [43.9]**	**<0.001 [85.2]**	0.483 [0.5]	0.124 [2.5]	0.098 [2.9]	0.171 [2.0]
<0.1 Hz	**0.006 [8.8]**	0.335 [1.0]	**<0.001 [76.8]**	0.535 [0.4]	0.227 [1.5]	0.172 [2.0]	0.360 [0.9]
>0.1 Hz	**0.009 [7.6]**	**<0.001 [96.4]**	**<0.001 [42.0]**	0.610 [0.3]	**0.042 [4.5]**	**0.016 [6.5]**	**0.008 [7.9]**
CTS_Old vs healthy controls							
Total	0.500 [0.5]	**<0.001 [32.3]**	**<0.001 [78.2]**	0.911 [0.0]	0.297 [1.1]	0.805 [0.1]	0.610 [0.3]
<0.1 Hz	0.597 [0.3]	**0.027 [5.5]**	**<0.001 [69.5]**	0.278 [1.2]	0.162 [2.1]	0.691 [0.2]	0.555 [0.4]
>0.1 Hz	0.430 [0.6]	**<0.001 [49.8]**	**<0.001 [34.2]**	0.465 [0.5]	0.071 [3.5]	0.262 [1.3]	0.516 [0.4]
CTS_Young vs CTS_Old							
Total	0.103 [3.1]	**<0.001 [34.6]**	**<0.001 [46.3]**	0.469 [0.6]	0.579 [0.3]	0.301 [1.2]	0.144 [2.4]
<0.1 Hz	0.181 [2.0]	**0.045 [4.8]**	**<0.001 [44.6]**	0.082 [3.5]	0.497 [0.5]	0.524 [0.4]	0.226 [1.6]
>0.1 Hz	0.177 [2.0]	**<0.001 [64.6]**	**<0.001 [30.8]**	0.679 [0.2]	0.708 [0.1]	0.057 [4.3]	**0.045 [4.8]**

Repeated measures GLM ANOVA analysis of how the perturbed stance stability were affected by main factors “Chemotherapy” (denoted Chemo in the table) “Vision” and “Period” alone and by their main factor interactions

*In the CTS_Young vs CTS_Old GLM Anova evaluation the main factor “chemotherapy” represents the effect of receiving chemotherapy below 12 years of age (CTS_Young) vs from 12 years of age and older (CTS_Old).

**Table 3:** Stability changes between vibration period 1 and vibration period 4 when standing with Eyes Closed and Eyes Open.

Stability changes*	Vibration period 1 vs period 4	
	Eyes Closed	Eyes Open
Healthy controls		
Total	**<0.001 [−48]**	**<0.001 [−59]**
<0.1 Hz	**<0.001 [−64]**	**<0.001 [−73]**
>0.1 Hz	**<0.001 [−36]**	**0.002 [−29]**
CTS		
Total	**0.005 [−30]**	**0.001 [−48]**
<0.1 Hz	**0.006 [−40]**	**0.002 [−55]**
>0.1 Hz	0.058 [**−**22]	**0.005 [−40]**
CTS_Young		
Total	0.160 [**−**21]	**0.002 [−51]**
<0.1 Hz	0.232 [**−**29]	**0.004 [−60]**
>0.1 Hz	0.432 [**−**15]	0.084 [**−**41]
CTS_Old		
Total	**0.031 [−50]**	0.156 [**−**37]
<0.1 Hz	**0.031 [−65]**	0.438 [**−**36]
>0.1 Hz	0.063 [**−**38]	**0.031 [−37]**

^*^Stability changes as reflected by increased/decreased use of energy in percent are presented within the squared parenthesis.

**Table 4:** Subject characteristics, diagnosis and chemotherapy details.

Subject	Diagnosis	Gender	Age at treatment (years)	Age when assessed (years)	Brock’s hearing loss graders **	Chemotherapy treatment agents*
1	Sacrococcygeal teratoma	Female	0.1	23.4	0	Ble, Cis, Eto
2	Hepatoblastoma	Female	2.5	15.9	—	Adr, Cis
3	Embryonal teratoma	Female	2.5	17.7	2	Ble, Cis, Eto
4	Ewing sarcoma	Male	2.9	16.4	—	Act, Adr, Eto, Ifo, Vin
5	Ewing sarcoma	Female	8.6	30.0	—	Act, Adr, Ble, Cyc, Met, Vin
6	Neuroblastoma	Male	8.9	21.4	3	Car, Cis, Cyc, Eto, Mel, Vin
7	Immature teratoma	Female	9.1	18.5	0	Ble, Cis, Eto
8	Ewing sarcoma	Male	9.6	30.3	—	Act, Adr, Ble, Cyc, Met, Vin
9	Immature teratoma	Female	10.3	35.8	—	Act, Adr, Cyc, Vin
10	Ewing sarcoma	Male	10.7	33.1	3	Act, Adr, Ble, Cis, Cyc, Met, Vin
11	Osteosarcoma	Female	14.3	33.9	1	Act, Adr, Ble, Cis, Cyc, Met
12	Ewing sarcoma	Male	15.5	35.4	—	Act, Adr, Ble, Cyc, Met, Vin
13	Ewing sarcoma	Male	15.7	24.0	0	Adr, Cis, Ifo, Vin
14	Immature teratoma	Male	16.5	27.8	0	Ble, Cis, Eto
15	Ewing sarcoma	Male	16.8	23.7	0	Act, Adr, Cis, Cyc, Eto, Ifo, Vin
16	Osteosarcoma	Male	16.9	30.9	0	Adr, Cis, Met

^*^Act: Actinomycin-D; Adr: Adriamycin; Ble: Bleomycin; Car: Carboplatin; Cis: Cisplatin; Cyc: Cyclophosphamide; Eto: Etoposide; Ifo: Ifosfamide; Mel: Melphalan; Met: Methotrexate; Vin: Vincristine.

^**^Brock’s hearing loss grades:

0: <40 dB at all frequencies. 1: ≥40 dB at 8 kHz only, and <40 dB at all other frequencies. 2: ≥40 dB at 4 kHz and above, and <40 dB at all other frequencies. 3: ≥40 dB at 2 kHz and above, and <40 dB at all other frequencies. 4: ≥40 dB at 1 kHz and above, and <40 dB at all other frequencies.
